# Efficacy of Feed-Based Genome-Free Bacterial Vaccine Against *Aeromonas hydrophila* Infection in Red Tilapia (*Oreochromis* sp.)

**DOI:** 10.3390/vaccines12111271

**Published:** 2024-11-11

**Authors:** Nur Shidaa Mohd Ali, Mohamad Syazwan Ngalimat, Boon Chuan Lim, Chia-Chen Hsu, Annas Salleh, Muhammad Farhan Nazarudin, Ina Salwany Md Yasin, Mohammad Noor Amal Azmai

**Affiliations:** 1 Laboratory of Aquatic Animal Health and Therapeutics, Institute of Bioscience, Universiti Putra Malaysia, Serdang 43400, Selangor, Malaysia; gs60111@student.upm.edu.my (N.S.M.A.); m_farhannaza@upm.edu.my (M.F.N.); salwany@upm.edu.my (I.S.M.Y.); 2 Department of Microbiology, Faculty of Biotechnology and Biomolecular Sciences, Universiti Putra Malaysia, Serdang 43400, Selangor, Malaysia; gs58188@student.upm.edu.my; 3 Oxford SimCell Ltd., Centre for Innovation and Enterprise, Begbroke Science Park, Begbroke, Oxfordshire OX5 1PF, UK; boon.lim@oxfordsimcell.com (B.C.L.); jane.hsu@oxfordsimcell.com (C.-C.H.); 4 Laboratory Diagnosis, Department of Veterinary, Faculty of Veterinary Medicine, Universiti Putra Malaysia, Serdang 43400, Selangor, Malaysia; annas@upm.edu.my; 5 Department of Aquaculture, Faculty of Agriculture, Universiti Putra Malaysia, Serdang 43400, Selangor, Malaysia; 6 Department of Biology, Faculty of Science, Universiti Putra Malaysia, Serdang 43400, Selangor, Malaysia

**Keywords:** Tilapia, *Aeromonas hydrophila*, genome-free bacteria, feed-based vaccine, immunity

## Abstract

*Aeromonas hydrophila* causes motile *Aeromonas* septicemia (MAS), a disease with a high mortality rate in tilapia culture. Feed-based vaccines with the incorporation of inactivated whole-cell bacteria into the feed offer promising tools to control MAS. Currently, the incorporation of genome-free bacteria as bacterial vaccine through the implementation of SimCells^®^ technology into the feed has become a particular interest. **Background/Objectives**: This study investigates the efficacy of a feed-based vaccine incorporating genome-free *A. hydrophila* (FBV-GFAH) against MAS infection in red tilapia. **Methods**: The vaccine was prepared and delivered at 5% fish body weight for three consecutive days in weeks 0 (prime vaccination) and 2 (first booster vaccination), orally. Throughout a five-week experimental period, the immune-related genes (IL-1*β*, MHC-II, CD4, IgT, and IgM) expression in the hindgut and head kidney of the fish was determined using RT-qPCR assay. Lysozyme (serum) and overall IgM (serum, gut lavage, and skin mucus) productions were also detected. **Results**: Fish vaccinated with FBV-GFAH showed a significant (*p* ≤ 0.05) improvement in relative percent survival compared with unvaccinated fish following bacterial challenge. FBV-GFAH induced the expression of immune-related genes in the hindgut and head kidney, especially after booster vaccination. Furthermore, serum lysozyme activity and overall IgM production in serum, skin mucus, and gut lavage were also significantly (*p* ≤ 0.05) improved in the FBV-GFAH vaccinated fish than the unvaccinated fish. **Conclusions**: This study showed that FBV-GFAH is a promising feed-based vaccine technology to control MAS in cultured tilapia.

## 1. Introduction

Tilapia (*Oreochromis* spp.) have been cultivated all over the world over the past 80 years, and active production was documented in over 124 countries. Tilapia has been listed as the top five species of aquatic animal harvested globally in 2022, with a total production of 5.3 million tonnes, valued at about USD 160 million [1]. It is a fast-growing fish and hardy, as it is able to withstand a wide range of environmental conditions, including the high stocking densities during its culture [1]. Unfortunately, as the tilapia farming sector has grown, various pathogenic bacteria have led to recurrent disease outbreaks, which have caused the aquaculture industry to suffer substantial economic losses [2]. Among pathogenic bacteria, several *Aeromonas* spp., including *Aeromonas hydrophila,* have been reported to cause motile *Aeromonas* septicaemia (MAS) in tilapia culture [3,4,5].

In Malaysia, mass mortality caused by MAS has been reported on tilapia culture due to infection of *A. hydrophila* [6]. *A. hydrophila* is a non-spore-forming, rod-shaped, Gram-negative, oxidase-positive, and facultative anaerobic bacteria. MAS is characterized by a broad range of clinical signs and symptoms in infected fish due to aeromonad toxins [6]. Typically, tilapia with MAS lose their normal balances, and infected fish gasp at the surface, and are lethargic. MAS may be acute, chronic, or latent, and the clinical signs of infected fish include hemorrhages, ulcerations, abscesses, exophthalmia, abdominal distension, and blackened skin [7,8]. Meanwhile, the internal signs of infected fish include enlarged gall bladder, pale liver, and hemorrhages of the kidney [6,9,10]. In fact, the Food and Agriculture Organization of the United Nations has listed *A. hydrophila* as one of the important bacterial diseases of tilapia, including their zoonotic potential and risk of antimicrobial resistance [10].

Vaccines are regarded as an effective method to control bacterial diseases. Feed-based vaccines, which are developed by incorporating inactivated whole-cell bacteria directly into fish feed, have been considered a promising alternative to control bacterial diseases [11]. Vaccination with a feed-based bivalent vaccine incorporating formalin-killed whole-cell of *A. hydrophila* and *Streptococcus agalactiae* mixed with 10% palm oil in red hybrid tilapia has shown improving relative percent survival [12] and fish immuno-transcriptomic responses [13]. Numerous studies have also indicated the efficacy of feed-based vaccines incorporating formalin-killed whole-cell bacteria [14]. However, the protection induced by genome-free bacterial cells as bacterial vaccine in aquaculture is still new and limited. In fact, the limitation of formalin inactivation potentially alters the integrity of the bacterial whole-cell, thus affecting the protective effect of the vaccine [15]. To achieve a relatively good immune effect through oral immunization, the incorporation of genome-free bacterial cells as vaccine candidate into the fish feed warrants scientific investigation.

The genome-free bacterial cells, also known as simple cells (SimCells^®^; Oxford SimCell Ltd., Oxfordshire, UK) possess mainly three features: (i) cannot replicate but otherwise resemble their living counterparts; (ii) safe; and (iii) immunogenic [16]. The genome-free bacterial cell production process involves transforming the parent bacteria with a plasmid encoding the homing endonuclease *IceuI*, which creates double-strand breaks in the host genome. The genome fragments are subsequently degraded by native cellular machinery, resulting in genome-free bacterial cell production [17]. Noteworthy, mice administered with genome-free bacterial cells exhibited no signs of ill health and produced higher levels of specific antibodies compared to those receiving UV-irradiated bacteria, suggesting the convenient uses of genome-free bacterial cells as a safe and immunogenic bacterial vaccine [18].

This study aims to investigate the efficacy of a feed-based vaccine incorporating genome-free *A. hydrophila* (FBV-GFAH) against MAS in red tilapia (*Oreochromis* sp.). The efficacy and immunogenicity of FBV-GFAH were assessed in vivo in red tilapia. As the uses of genome-free bacterial vaccine in aquaculture are poorly investigated, this study reported herein provides a helpful reference for the use of feed-based genome-free bacterial vaccines against MAS in the tilapia farming industry.

## 2. Materials and Methods

### 2.1. Source of Bacteria

*Aeromonas hydrophila* strain Ah1sa5 (GenBank: OR462201.1), which was initially isolated from infected red hybrid tilapia [19], was subjected to genome-free synthesis. The synthesis of genome-free *A. hydrophila* was conducted by Oxford SimCell Ltd., Oxfordshire, UK. The genome-free *A. hydrophila* was synthesized according to the established study [20]. Briefly, *A. hydrophila* was conjugated with SimCell^®^ plasmid from *Escherichia coli*. Prior to conjugation, *A. hydrophila* and *E. coli* were grown on tryptone soya broth (TSB; Oxoid, Hampshire, UK) and incubated overnight at 30 °C with shaking at 180 rpm. Next, 1 mL of each bacterial culture was washed in 1× phosphate-buffered saline (PBS) three times through centrifugation (8000× *g*, 15 min at 4 °C) and further resuspended in 500 µL of PBS. The *E. coli* and *A. hydrophila* suspensions were mixed to the final volume of 100 µL. The mixture was cultured on TSA supplemented with 2,6-diaminopimelic acid and incubated for 18 h at 30 °C. The grown bacterial colony was transferred into 100 µL of PBS and further cultured on TSA Kan150 overnight at 30 °C. The presence of SimCell^®^ plasmid in transformed *A. hydrophila*-SimCells^®^ was successfully verified by PCR.

### 2.2. Feed-Based Vaccine Preparation

**Figure 1:**
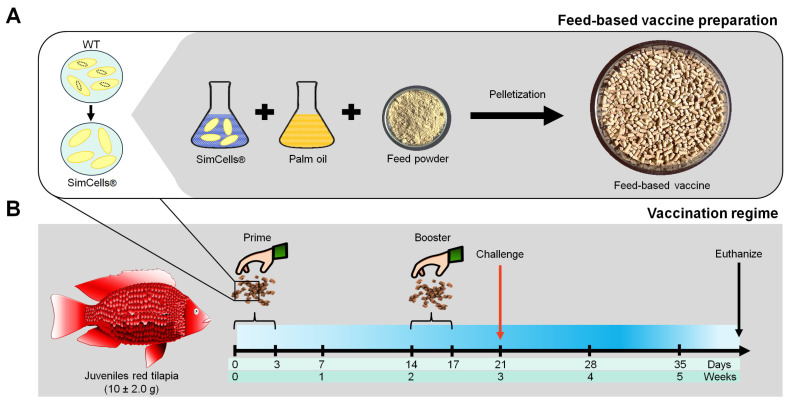
Illustration of feed-based vaccine preparation and vaccination regime. (**A**) The preparation of a feed-based vaccine incorporating genome-free bacteria. The wild-type (WT) strain of *A. hydrophila* was synthesized to be genome-free, also known as simple cells (SimCells^®^). A feed-based vaccine was prepared by incorporating *A. hydrophila*-SimCells^®^ mixed with palm oil and feed powder before pelletization. (**B**) The feed-based vaccine was delivered at 5% fish body weight for three consecutive days on days 0–3, followed by booster vaccination on days 14–17. The challenge test was conducted on day 21. For immunological analyses, the hindgut, head kidney, serum, gut lavage, and skin mucus samples were collected at weekly intervals from week 0 to week 5. During vaccination weeks (weeks 0 and 2), samples were collected 24 h after vaccination for three days.

To prepare a feed-based vaccine, the FBV-GFAH was generated by mixing the genome-free *A. hydrophila* with 10% (*v*/*w*) commercial food-grade palm oil and commercial tilapia feed powder according to the MyIPO Malaysia, patent number PI20222001807 (Figure 1A). The nutritional composition and ratio of the commercial tilapia feed used in this study were recorded for carbohydrate (44.26 ± 0.15%), energy (354.00 ± 0.00%), moisture (7.87 ± 0.02%), protein (34.36 ± 0.02%), total ash (9.17 ± 0.02%), and total fat (4.36 ± 0.02%).

Briefly, 100 mL of genome-free *A. hydrophila* at approximately 1 × 10^12^ CFU/mL in 1 × PBS at pH 7 was homogenized with 100 mL of palm oil. Next, 1 × PBS was added to the solution mixture until the total volume reached 1 L. To form pellets, 1 L of solution mixture and 1 kg of commercial tilapia feed powder were mixed uniformly in a mixer machine and pelletized using a pelleting machine (Golden Avill, Guangdong Province, China). The feed-based vaccine prepared by incorporating formalin-killed *A. hydrophila* strain Ah1sa5 (FBV-FKAH) at a similar concentration was used as a positive control. Meanwhile, the feed-based vaccine prepared without the addition of bacteria was used as a negative control.

All feed-based vaccines were oven-dried for 12 h at 28 °C and stored at room temperature (25 ± 2 °C). The effects of FBV-GFAH, FBV-KFAH, and negative control vaccines on the growth performance of red tilapia were analyzed according to the previous study [12].

### 2.3. Fish Vaccination and Challenge Experiments

This study was performed for 35 days of duration. The water quality throughout the experimental period was recorded at 25.0 ± 0.5 °C for temperature, 6.0 ± 1.0 mg/L for dissolved oxygen, 7.0 ± 1.0 for pH, and 0.010 ± 0.001 mg/L for ammonia-nitrogen. The experiment related to fish handling followed the Malaysian Code of Practice for the Care and Use of Animals for Scientific Purposes and was approved by the Institutional Animal Care and Use Committee, Universiti Putra Malaysia, with the approval number of UPM/IACUC/AUP-R024/2024.

After the acclimatization process, red tilapia (*n* = 360) with mean body weight at 10.0 ± 2.0 g were separated into three groups (Group 1: unvaccinated fish as a negative control; Group 2: fish vaccinated with FBV-FKAH as a positive control; Group 3: fish vaccinated with FBV-GFAH) with triplicates (40 fish/replicate). Prior to vaccination, fish have been starved for 24 h. The vaccine was delivered at 5% of fish body weight for three consecutive days on days 0–3, followed by boosters on days 14–17 (Figure 1B) according to the previous study [12]. Fish were fed with commercial tilapia feed twice daily for the non-vaccination days.

On day 21, 30 fish individuals from each group were transferred into new tanks and challenged by intraperitoneal injection with 4.0 × 10^4^ LD_50_/mL of live *A. hydrophila* strain Ah1sa5 [12]. All challenged fish were observed and recorded daily for 14 days for any abnormal symptoms.

The mortality and relative percentage survival (RPS) were observed daily for 14 days after bacterial challenge. The mortality was calculated: mortality (%) = (number of fish dead/total number of fish) × 100. The RPS was calculated: RPS = 100 − (average vaccinated fish mortality/average control fish mortality × 100).

Subsequently, the PCR assay using REdiant Master Mix (1st BASE, JTC MedTech Hub, Singapore) was used to confirm the *A. hydrophila* infection in death-challenged fish organs using species-specific primers for the amplification of gyrB gene (forward primer: 5′-TCCGGCGGTCTGCACGGCGT-3′ and reverse primer 5′-TTGTCCGGGTTGTACTCGTC-3′) [21]. The gyrB gene encodes *β* subunit of DNA gyrase, a type-II DNA topoisomerase that has been identified as a useful phylogenetic marker for *A. hydrophila*. The gyrB gene was amplified according to the established thermal cycling condition [13].

### 2.4. Immunological Assessments

The immunological assessments throughout the vaccination trial were investigated based on the immune-related gene expression in the hindgut and head kidney samples using an RT-qPCR assay. Briefly, the samples (6 fish/groups) were collected from week 0 to week 5 (Figure 1B) at weekly intervals. During vaccination weeks (weeks 0 and 2), samples were collected 24 h after vaccination for three days. Fish was anesthetized with MS-222 at a dose of 105 mg/L before sample collection. The hindgut and head kidney samples have been collected according to the previous study [13].

The samples were immersed into RNAlater^®^ Tissue Collection (Invitrogen, Carlsbad, CA, USA) and stored at −80 °C before being subjected to total RNA extraction using TRIzol™ reagent (Invitrogen, CA, USA). The obtained total RNA were reverse transcribed to cDNA using a OneScript^®^ Hot cDNA Synthesis Kit (Applied Biological Materials Inc., Richmond, BC, Canada). The QuantiNova™ SYBR^®^ Green PCR kit (QIAGEN, Hilden, Germany) and Rotor-Gene RT-qPCR (QIAGEN, Hilden, Germany) were used to perform the qPCR assay according to the established thermal cycling condition [13]. The immune-related genes (IL-1*β*, MHC-II, CD4, IgT, and IgM) expression was evaluated, while *β*-actin (ACTB) was used as a reference gene (Table 1) as this gene expressed stably in the fish gut and head kidney.

For expression analysis, the differences between the cycle threshold (CT) means of immune-related genes and the means of reference gene (ACTB) in the vaccinated and control groups were determined. The relative expression level of each gene was calculated; relative expression level = 2^−ΔΔCT^, where ΔΔCT = [CT immune-related gene (vaccinated group) − CT reference gene (vaccinated group)] − [CT immune-related gene (control group) − CT reference gene (control group)].

The serum lysozyme activity and overall IgM production in serum, gut lavage, and skin mucus were also investigated. Serum, gut lavage, and skin mucus samples (6 fish/groups) were collected from week 0 to week 5, according to the previous study [12], at weekly intervals. During vaccination weeks (weeks 0 and 2), samples were collected 24 h after vaccination for three days. The lysozyme activity in serum was measured using an EnzChek™ Lysozyme Assay Kit with CAT. NO. E22013 (Invitrogen, CA, USA). Meanwhile, overall IgM production in serum, gut lavage, and skin mucus was measured using a Fish Immunoglobulin M ELISA Kit with CAT. NO. MBS042385 (sensitivity: 5.0 µg/mL, detection range: 25–800 µg/mL), following the manufacturer’s instructions (MyBioSource Company, San Diego, CA, USA).

### 2.5. Statistical Analysis

The statistical data analyses comparing each group’s results were carried out using SAS 9.3 software (SAS Institute Inc., Cary, NC, USA), according to a one-way ANOVA with a Tukey-Kramer post hoc test. A value of *p* ≤ 0.05 was considered statistically significant.

## 3. Results

### 3.1. Vaccines Efficacy and Comparison

**Figure 2:**
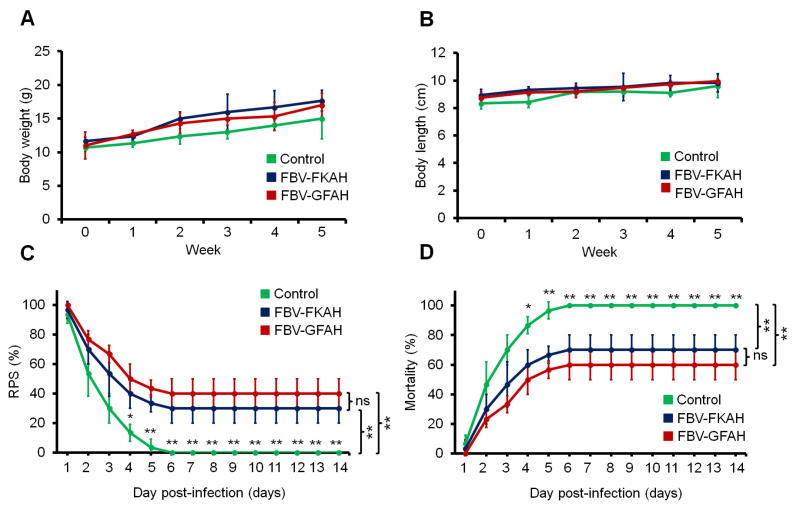
The efficacy of a feed-based genome-free bacterial vaccine in red tilapia. The effect of vaccination on (**A**) body weight and (**B**) body length of red tilapia. (**C**) The relative percentage survival (RPS) and (**D**) mortality of vaccinated fish hour post-infection with *A. hydrophila* strain Ah1sa5. Results are in x ± SD (error bar), *n* = 3. Statistical differences between treatments using one-way ANOVA with a Tukey-Kramer post hoc test: * = *p* ≤ 0.05, ** = *p* ≤ 0.01, and ns = not significant. Note: Control: feed-based vaccine without the addition of bacteria, served as a negative control; FBV-FKAH: feed-based vaccine incorporating formalin-killed *A. hydrophila* strain Ah1sa5, served as a positive control; and FBV-GFAH: feed-based vaccine incorporating genome-free *A. hydrophila*.

Fish vaccinated with FBV-GFAH and FBV-KFAH did not show any significant difference (*p* ≥ 0.05) in fish body weight (Figure 2A) and length (Figure 2B) compared to the unvaccinated fish throughout the five-week experimental period. At 14 days (336 h) post challenged with the pathogenic *A. hydrophila* strain Ah1sa5, the RPS (Figure 2C) and mortality (Figure 2D) were analyzed. Fish vaccinated with FBV-GFAH and FBV-KFAH showed a significant improvement (*p* ≤ 0.05) in RPS compared with unvaccinated fish. The highest RPS was detected in fish vaccinated with FBV-GFAH (RPS at 40 ± 10%), followed by FBV-KFAH (RPS at 30 ± 10%) and unvaccinated fish (RPS at 0 ± 0%).

**Figure 3:**
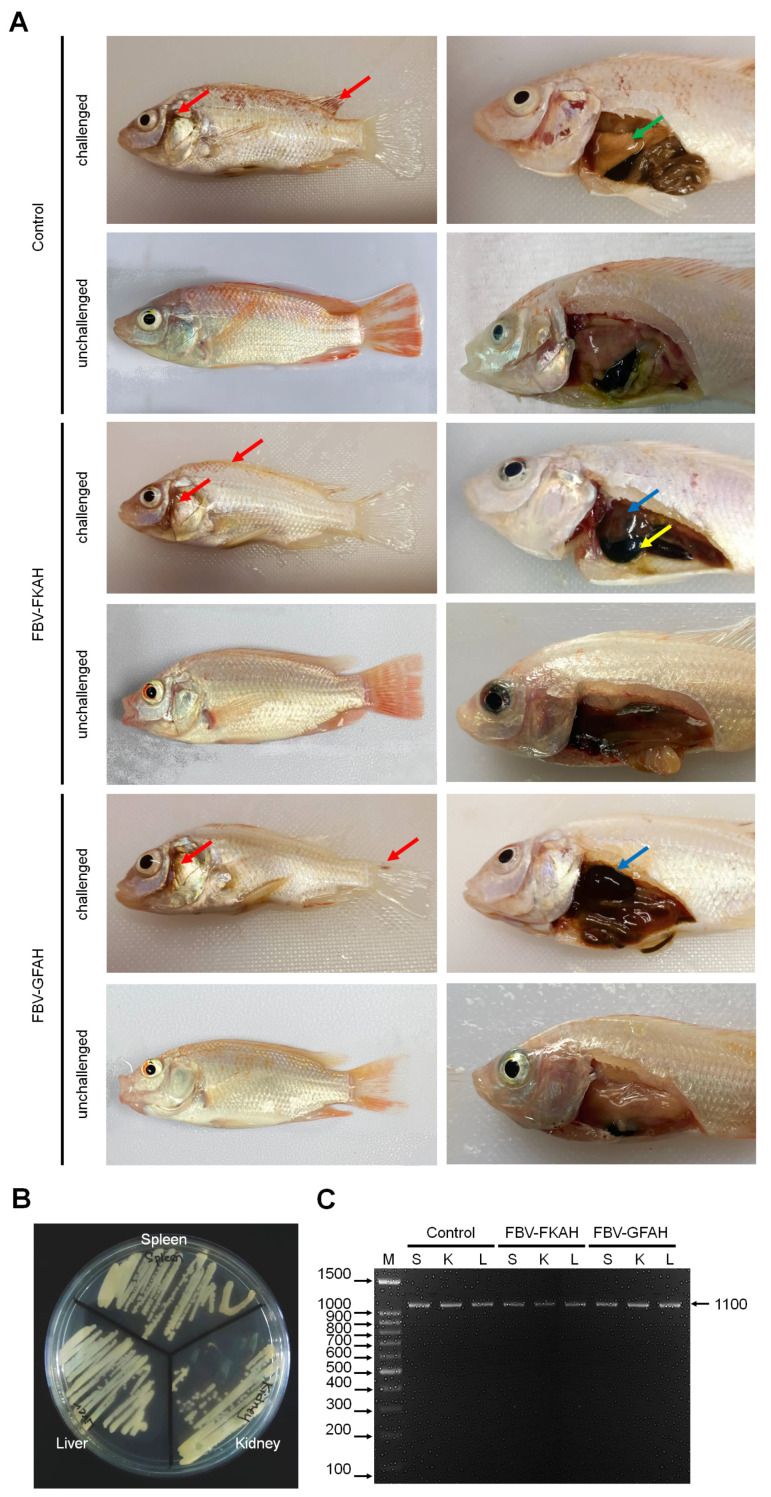
The challenge test. (**A**) Clinical signs and gross lesions following the challenge test. All treatment groups showed similar external lesions, including haemorrhages (red arrows) in the operculum skin and dorsal, caudal, and pectoral fins. Internal lesions such as pale and enlarged liver (green arrow), swollen liver (blue arrows), and swollen gall bladder (yellow arrow) were also observed. (**B**) Isolation of *A. hydrophila* strain Ah1sa5 from the infected dead fish organs. (**C**) The PCR detection of *A. hydrophila* strain Ah1sa5 at 1100 bp from the spleen (S), kidney (K), and liver (L) of infected dead fish using species-specific primers. Note, M = 100 bp DNA ladder (1st BASE, JTC MedTech Hub, Singapore); Control: feed-based vaccine without the addition of bacteria, served as a negative control; FBV-FKAH: feed-based vaccine incorporating formalin-killed *A. hydrophila* strain Ah1sa5, served as a positive control; and FBV-GFAH: feed-based vaccine incorporating genome-free *A. hydrophila*.

Typical clinical signs of *A. hydrophila* infection were observed in the morbid and dead fish from all treatment groups (Figure 3A). No other pathogen than *A. hydrophila* was isolated from the spleen, kidney, and liver of infected dead fish (Figure 3B). The presence of *A. hydrophila* in infected fish organs from all treatment groups was successfully detected according to the PCR assay, with the amplification band at 1100 bp (Figure 3C).

### 3.2. Immune-Related Gene Expression

**Figure 4:**
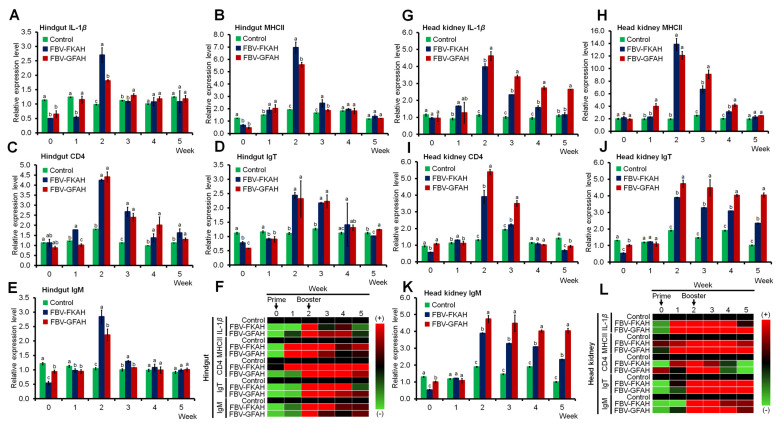
Immune-related gene expression in the hindgut and head kidney. The expression of (**A**) IL-1*β*, (**B**) MHCII, (**C**) CD4, (**D**) IgT, and (**E**) IgM normalized with ACTB in the hindgut. (**F**) The heatmap of immune-related gene expression in the hindgut of vaccinated fish relative to control fish. The expression of (**G**) IL-1*β*, (**H**) MHCII, (**I**) CD4, (**J**) IgT, and (**K**) IgM normalized with ACTB in the head kidney. (**L**) The heatmap of immune-related gene expression in the head kidney of vaccinated fish relative to control fish. Results are in x ± SD (error bar), *n* = 3, where different superscript letters differ significantly at *p* ≤ 0.05. For heatmaps, data are presented as percentage control (%) = vaccinated/control × 100, assuming control is 100%. Note: Control: feed-based vaccine without the addition of bacteria, served as a negative control; FBV-FKAH: feed-based vaccine incorporating formalin-killed *A. hydrophila* strain Ah1sa5, served as a positive control; and FBV-GFAH: feed-based vaccine incorporating genome-free *A. hydrophila*.

The effect of vaccination on the immune-related gene expression in the mucosal (hindgut) and systemic (head kidney) organs has been investigated by RT-qPCR assay. The transcription of IL-1*β* (Figure 4A), MHCII (Figure 4B), CD4 (Figure 4C), IgT (Figure 4D), and IgM (Figure 4E) normalized with ACTB in the hindgut were determined. The results showed that all examined genes were significantly (*p* ≤ 0.05) up-regulated in the hindgut of fish vaccinated with FBV-GFAH and FBV-KFAH when compared with the unvaccinated fish, especially after booster vaccination in week 2. Additionally, the heatmap analysis has found that the highest expression of all examined genes in the hindgut was detected from week 2 to week 4 (Figure 4F). The expression of all examined genes in the hindgut started to reduce in week 5.

The transcription of IL-1*β* (Figure 4G), MHCII (Figure 4H), CD4 (Figure 4I), IgT (Figure 4J), and IgM (Figure 4K) in the head kidney when normalized with ACTB also showed a similar pattern as in the hindgut. All analyzed genes were up-regulated in the head kidney of fish vaccinated with FBV-GFAH and FBV-KFAH when compared to unvaccinated fish, especially after booster vaccination in week 2 (Figure 4L).

### 3.3. Serum Lysozyme Production

**Figure 5:**
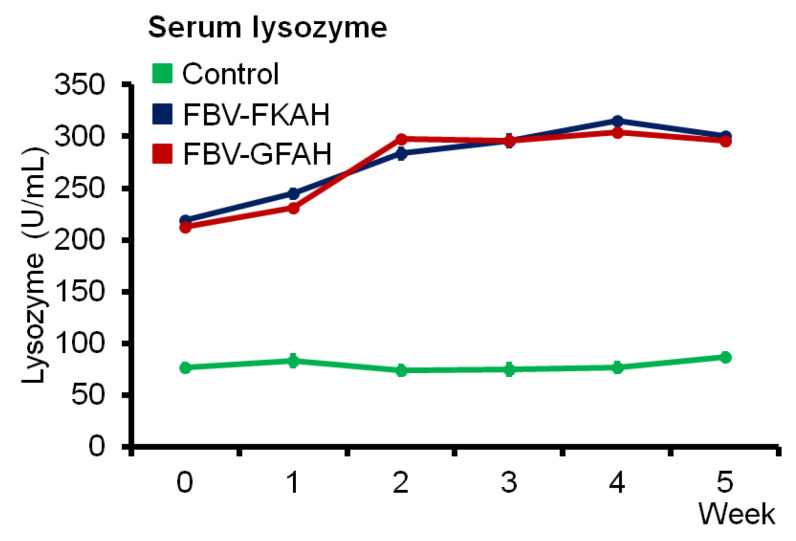
Serum lysozyme productions in red tilapia vaccinated with a feed-based genome-free bacterial vaccine. Results are in x ± SD (error bar), *n* = 3. Note: Control: feed-based vaccine without the addition of bacteria, served as a negative control; FBV-FKAH: feed-based vaccine incorporating formalin-killed *A. hydrophila* strain Ah1sa5, served as a positive control; and FBV-GFAH: feed-based vaccine incorporating genome-free *A. hydrophila*.

Serum lysozyme production in fish vaccinated with FBV-GFAH and FBV-FKAH showed that the lysozyme increased after booster vaccination in week 2 (Figure 5). The production of lysozyme in fish serum in both vaccinated groups remained stable until week 4 post-vaccination, and then the production started to reduce in week 5. A significantly higher level (2–3 times) of serum lysozyme production was detected in fish vaccinated with FBV-GFAH and FBV-FKAH when compared with the unvaccinated fish (*p* ≤ 0.05). However, no significant serum lysozyme production pattern was observed between fish vaccinated with FBV-GFAH and FBV-FKAH.

### 3.4. Immunoglobulin M (IgM) Production

**Figure 6:**
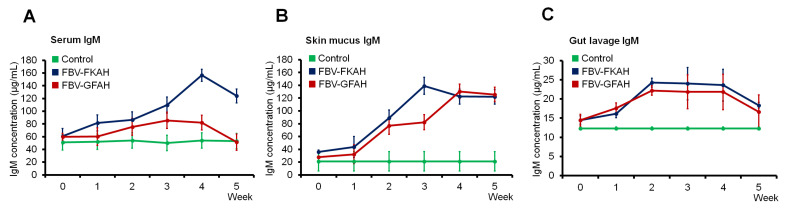
Immunoglobulin M (IgM) production in red tilapia vaccinated with a feed-based genome-free bacterial vaccine. (**A**) Serum, (**B**) skin mucus, and (**C**) gut lavage overall IgM production. Results are in x ± SD (error bar), *n* = 3. Note: Control: feed-based vaccine without the addition of bacteria, serving as a negative control; FBV-FKAH: feed-based vaccine incorporating formalin-killed *A. hydrophila* strain Ah1sa5, served as a positive control; and FBV-GFAH: feed-based vaccine incorporating genome-free *A. hydrophila*.

The overall IgM production in the serum (Figure 6A), skin mucus (Figure 6B), and gut lavage (Figure 6C) of vaccinated and unvaccinated fish was determined. Fish vaccinated with FBV-GFAH and FBV-FKAH produced significantly (*p* ≤ 0.05) higher overall IgM production levels than unvaccinated fish, especially after the booster vaccination in week 2. Following oral administration of booster vaccination, the overall IgM production levels in fish vaccinated with FBV-GFAH and FBV-FKAH presented an incremental pattern and remained significantly high until week 4, when compared with unvaccinated fish.

For serum, fish vaccinated with FBV-FKAH showed the highest overall IgM production level at week 4 (156.38 ± 9.60 µg/mL), while fish vaccinated with FBV-GFAH showed the highest overall IgM production level at week 3 (85.61 ± 12.75 µg/mL). Fish vaccinated with FBV-FKAH showed the highest overall IgM production level in skin mucus at week 3 (139.26 ± 13.39 µg/mL), while fish vaccinated with FBV-GFAH showed the highest overall IgM production level at week 4 (130.52 ± 11.96 µg/mL). It has been found that the highest overall IgM production level in gut lavage of fish vaccinated with FBV-FKAH and FBV-GFAH was detected at week 2, with overall IgM production at 24.24 ± 1.17 µg/mL and 22.20 ± 1.26 µg/mL, respectively. The overall IgM production levels in serum, skin mucus, and gut lavage of fish vaccinated with FBV-GFAH and FBV-FKAH started to reduce in week 5.

## 4. Discussion

In this work, the genome-free, also known as simple cells (SimCells^®^) of *A. hydrophila* was incorporated into the fish feed with the intention of improving the red tilapia immunity against MAS. The genome-free bacteria have been proven to be a safe agent (unable to replicate) for synthetic biology applications [16]. Genome-free bacteria are unable to replicate due to the absence of chromosomes, reducing their biosafety concerns related to genetically modified microorganisms and the potential of horizontal gene transfer within bacteria [16,25].

Unlike inactivated bacterial cells using chemicals such as formalin, genome-free bacterial cells retain their original bacterial cell membrane. This has alleviated its potential as a safe and immunogenic bacterial vaccine [18]. In this study, it has been proven that the FBV-GFAH had no negative effect on the fish’s growth performance. Additionally, the FBV-GFAH was found to improve the mucosal and systemic immunities of red tilapia, as indicated through immune-related gene expression analysis.

The efficacy of a vaccine is indicated based on the ability of vaccine antigens to stimulate innate and adaptive immunological responses [12]. The effectiveness of a vaccine-induced tilapia’s immune response has been proven in both mucosal (hindgut) and systemic (spleen and head-kidney) organs of fish vaccinated with an oral bivalent vaccine incorporating formalin-killed whole-cell of *A. hydrophila* and *Streptococcus iniae* [26]. The expression of IL-1*β*, MHCII, CD4, and IgT in the hindgut, spleen, and head-kidney of vaccinated fish was found to remain significantly higher (*p* ≤ 0.05) than the unvaccinated fish [26]. Hence, to understand the immunological basis of the FBV-GFAH efficacy, the relative expression of immune-related genes, including IL-1*β*, MHCII, CD4, IgT, and IgM, in the mucosal (hindgut) and systemic (head kidney) organs was investigated.

IL-1*β* is a pro-inflammatory cytokine involved in the early innate immunological response that is induced by activated immune cells upon stimulation by infectious agents [27]. It plays a role in immune cell recruitment and activation, pro-inflammatory cytokine production, and adaptive immunity modulation [28]. The expression of IL-1*β* in vaccinated fish upon vaccination was reported in various fish species, including Nile tilapia [13,26,29], zebrafish [30], Asian seabass [31], grouper [32] and yellow croaker [33]. In this study, higher expression of IL-1*β* was detected in the hindgut and head kidney of fish vaccinated with FBV-GFAH, especially after booster vaccination in week 2, suggesting an early induction of innate immunological response [26]. The expression of IL-1*β* showed an incremental pattern in the hindgut and head kidney of fish vaccinated with FBV-GFAH between prime (week 0) and booster (week 2) vaccinations. It is believed that the stimulation by infectious agents from FBV-GFAH after vaccination in week 0 has initiated the expression of IL-1*β*. Upon recognition of infectious agents, the expression of IL-1*β* was further improved as detected after booster vaccination in week 2.

Additionally, the adaptive immunological response depends on the presentation of antigens by major histocompatibility complex (MHC) markers available on antigen-presenting cells (APCs) [34]. CD4 plays a role in the immunological response by helping to present antigens to CD4+ T lymphocytes [35]. Meanwhile, MHCII is a T-cell co-receptor that can be found on APCs associated with antigen recognition [36]. Noteworthy, high expression of CD4 and MHCII has been reported in vaccinated fish organs, including the tilapia’s hindgut, spleen, and head-kidney [26] and the flounder’s intestines, spleen, and kidneys [34]. Interestingly, significant (*p* ≤ 0.05) induction of CD4 and MHCII immune-related genes was detected in the hindgut and head kidney of fish vaccinated with FBV-GFAH, illustrating the interaction of mucosal and systemic immune cells in inducing the adaptive immunological response.

The adaptive immunological response was also correlated with immunoglobulins (Igs) expression [37]. The commonly studied Igs identified in fish include IgT and IgM, in which IgT is prominent in the mucosal system and IgM is prevalent in the systemic system [37]. As confirmed using the RT-qPCR assay, the IgT and IgM expression in the hindgut and head kidney of fish vaccinated with FBV-GFAH was significantly (*p* ≤ 0.05) induced after booster vaccination in week 2, indicating that Igs potentially play a role in protecting fish from pathogenic antigens. Additionally, up-regulation of Igs was also reported in red hybrid tilapia vaccinated with a feed-based vaccine incorporating the formalin-killed whole-cell of *A. hydrophila* [12]. Overall, results suggested that the FBV-GFAH has successfully stimulated mucosal and systemic immunities in red tilapia, as proven by the expression of immune-related genes following oral vaccination.

To further understand the efficacy of FBV-GFAH in improving the red tilapia immune response, the production of lysozyme and overall IgM in vaccinated fish has also been analyzed. It has been reported that the immuno-efficacy of the vaccine was detected based on the production of lysozyme and IgM [24,35,38]. In a previous study, a feed-based vaccine incorporating formalin-killed whole-cell of *A. hydrophila* and *S. agalactiae* was found to stimulate immunological responses in red hybrid tilapia by the production of lysozyme and IgM [12]. In this study, a significantly (*p* ≤ 0.05) higher level of serum lysozyme production was detected in fish vaccinated with FBV-GFAH compared with the unvaccinated fish. Fish vaccinated with FBV-GFAH also showed significantly (*p* ≤ 0.05) higher overall IgM production levels than unvaccinated fish, especially after the booster vaccination in week 2. Even though the overall production of IgM has been successfully proven, the production of specific IgM against *A. hydrophila* needs to be investigated [12]. The results suggested that vaccination with FBV-GFAH in weeks 0 (prime vaccination) and 2 (first booster vaccination) has improved lysozyme and overall IgM production in red tilapia. However, it has been found that the production of lysozyme and overall IgM started to reduce in week 5, suggesting the need for additional vaccination boosters to further prolong the fish’s immunity [12].

Overall, the uses of genome-free bacteria in aquaculture as a bacterial vaccine are still new and limited. This study has demonstrated the insignificant (*p* ≥ 0.05) pattern of RPS in fish vaccinated with FBV-GFAH compared to the FBV-FKAH that served as a positive control. However, the RPS obtained in this study is only 30% for fish vaccinated with FBV-KFAH and 40% for fish vaccinated with FBV-GFAH. To note, studies using a single booster dosage provided RPS around 20–40% [39,40]. It is believed that additional booster dosages are needed to further improve the RPS. Additionally, the induction of mucosal and systemic immunities in fish vaccinated with FBV-GFAH also showed an overall similar pattern with the positive control group. The advantages of genome-free bacteria as a safe and immunogenic bacterial vaccine have offered a convenient alternative to replace the use of chemically inactivated bacteria as bacterial vaccines [16]. With the advantages of genome removal and being unable to replicate, genome-free bacteria are potentially able to reduce the risk of antimicrobial resistance development due to the lack of potential for horizontal gene transfer mechanisms within bacteria [16,26].

## 5. Conclusions

The results demonstrated that FBV-GFAH has the potential to induce mucosal and systemic immunities in red tilapia against MAS infection following oral administration. This study has suggested the immuno-efficacy of a genome-free bacteria as a bacterial vaccine in a feed-based vaccine. Moreover, as the uses of genome-free bacteria in aquaculture as a bacterial vaccine are still new and limited, this study offers an early benchmark for the potential application of feed-based vaccines incorporated with genome-free bacteria as a vaccine in the aquaculture industry.

## Figures and Tables

**Table 1:** Primers used for quantification of immune-related gene expression in *Oreochromis* sp. using RT-qPCR assay.

Gene Name	Gene Description	Sequence (5′ to 3′)	GenBank Accession Number	PCR Efficiency (%)	R-Squared (R²)	Product Size (bp)	Ref.
IL-1*β*	A pro-inflammatory cytokine involved in early innate immune response	F: CAAGGATGACGACAAGCCAACC	XM_003460625.2	107.52	0.9828	149	[22]
		R: AGCGGACAGACATGAGAGTGC					
MHCII	Play roles in adaptive immunity by helping to present antigens to CD4+ T lymphocytes	F: AGTGTGGGGAAGTTTGTTGGAT	JN967618.1	100.12	0.9846	207	[23]
		R: ATGGTGACTGGAGAGAGGCG					
CD4	A T-cell co-receptor that can be found on APCs associated with antigen recognition	F: TTCAGTGGCACTTTGCTCCTAA	XM031744220	98.88	0.9849	277	[23]
		R: TGGGCGATGATTTCCAACA					
IgT	Play roles in the defense mechanisms against pathogens in the mucosal compartments	F: GTGTCTGGTCTCCAGTCGTG	KY499641	91.39	0.9784	169	[24]
		R: TGTGCTCCACTTGTCCTTGG					
IgM	Play roles in host defense against pathogen infection	F: ACGAGGAAGCAGACTCAAGTTAT	XM_025906581.1	97.16	0.9975	175	[23]
		R: ACAATAGCTCTAGTTGTGTTAACC					
ACTB	A highly conserved protein with the function of producing filaments that form cross-linked networks in the cell cytoplasm	F: CCACACAGTGCCCATCTACGA	EU887951.1	99.23	0.9835	111	[22]
		R: CCACGCTCTGTCAGGATCTTCA					

Note, abbreviations: IL-1*β* = interleukin-1*β*, MHCII = major histocompatibility complex class II, CD4 = cluster of differentiation 4, APCs = antigen-presenting cells, IgT = immunoglobulin T, IgM = immunoglobulin M, ACTB = *β*-actin, F = forward primer, and R = reverse primer.

## Data Availability

Data are contained within the article.
